# Wavelet coherence as a measure of trunk stabilizer muscle activation in wheelchair fencers

**DOI:** 10.1186/s13102-021-00369-y

**Published:** 2021-10-30

**Authors:** Monika Błaszczyszyn, Zbigniew Borysiuk, Katarzyna Piechota, Krzysztof Kręcisz, Dariusz Zmarzły

**Affiliations:** 1 Faculty of Physical Education and Physiotherapy, Opole University of Technology, Prószkowska 76, 45-758 Opole, Poland; 2 Faculty of Electrical Engineering, Automatics and Computer Science, Opole University of Technology, Prószkowska 76, 45-758 Opole, Poland

**Keywords:** Wavelet analysis, Para-athletes, Spinal cord injury, Electromyography, Frequency levels

## Abstract

**Background:**

Intermuscular synchronization constitutes one of the key aspects of effective sport performance and activities of daily living. The aim of the study was to assess the synchronization of trunk stabilizer muscles in wheelchair fencers with the use of wavelet analysis.

**Methods:**

Intermuscular synchronization and antagonistic EMG–EMG coherence were evaluated in the pairs of the right and the left latissimus dorsi/external oblique abdominal (LD/EOA) muscles. The study group consisted of 16 wheelchair fencers, members of the Polish Paralympic Team, divided into two categories of disability (A and B). Data analysis was carried out in three stages: (1) muscle activation recording using sEMG; (2) wavelet coherence analysis; and (3) coherence density analysis.

**Results:**

In the Paralympic wheelchair fencers, regardless of their disability category, the muscles were activated at low frequency levels: 8–20 Hz for category A fencers, and 5–15 Hz for category B fencers.

**Conclusions:**

The results demonstrated a clear activity of the trunk muscles in the wheelchair fencers, including those with spinal cord injury, which can be explained as an outcome of their intense training. EMG signal processing application have great potential for performance improvement and diagnosis of wheelchair athletes.

## Background

Wheelchair athletes make extensive use of arm and trunk movements which alter their center of mass and activate compensatory responses to maintain stability in the sitting position. This is a conformation that practicing adapted sports can affect trunk muscle activation [1]. Wheelchair users always require properly coordinated movements of the upper limbs and the trunk. Such motor behavior significantly changes their center of mass and requires adaptive reactions in order to maintain a stable sitting posture [2–4]. Surface electromyography (sEMG) can be used to accurately identify the considerable electrical activity of trunk muscles while performing tasks with varying degrees of difficulty [5]. However, there has been no evidence of significant differences in upper body muscle activity in wheelchair users with various neurological conditions. This is of particular importance for the qualification of people with disabilities in sport competitions.

The primary method for evaluating motor function in athletes with spinal cord injury is the American Spinal Injury Association Impairment Scale (ASIS), which tests muscle strength in five key muscles in each limb and sensory function. However, as emphasized by numerous authors, the approach to the use of ASIS is subjective, and the scale does not measure the activity of the trunk muscles [6–8]. Significant recovery can be achieved without an improvement in the ASIS grade, therefore prospective studies should incorporate more detailed neurological outcomes to prevent any potential underestimation of neurological recovery associated with the exclusive use of the ASIS scale [5, 6, 9]. Previous research has shown that injured people do not have effective control over muscle activation below the level of injury, and can acquire a new pattern of motor control aimed at maintaining posture. The new pattern can recruit muscles with intact innervation by altering core muscle recruitment [4, 10]. Surface EMG is a reliable tool for muscle activation assessment as well as for exploration of physiological processes accompanying muscle force generated during a movement. It can provide a sensitive technique for identifying trunk muscle activity in individuals with spinal cord injury [11, 12]. Coherence is a measure representing the degree of relationship between two time variable signals in the function of frequency. It usually takes the form of magnitude-squared coherence, and constitutes a mathematical function represented by real numbers [13]. Coherence analysis is an approach that can be applied for the purposes of assessing neuromuscular systems and gaining insights into the communication between the central and peripheral systems in motor activity control resulting from the evaluation of the corticospinal pathway activity [14]. Coherence analysis performed at different frequencies can offer valuable information regarding the functions of the nervous system in controlling muscle activity when various tasks are executed [15]. It is generally recognized that within the beta band, intermuscular coherence (15–30 Hz) takes its origin in the cortex. Despite the fact that this type of coherence has been investigated thoroughly for the beta waves, the nature and origins of intermuscular coherence continue to be investigated and questioned for lower ranges of frequencies [16]. A number of studies in physiology seek the application of muscle coherence estimation in indirect measurements of voluntary motor drive with regard to motor tasks. Several clinical studies on muscle coherence activity calculated within specific frequency bands have been conducted and they have contributed to the recognition of the activity of different neuronal systems [17–19]. The decomposition of frequency and temporally localized characteristics of wavelet transform provide data on the localized phase-frequency of relative signals. In biomechanical research the area of cross correlation has been successfully utilized to provide quantifying insight into temporal similarity between EMG signals, and to assess kinematic timing differences of joint structures [20–23].

The aim of the present study was to assess trunk stabilizer muscle activation in wheelchair fencers using coherence analysis. The analysis of trunk muscle coherence can provide important data indicating the diagnostic potential of the monitoring of trunk muscle activation at different frequency levels.

## Methods

### Participants

Sixteen members of the Polish Wheelchair Fencing Paralympic Team (7 from category A, 9 from category B) were selected to participate in the study. Category A comprises fencers after lower limbs amputation or with partial paresis, having freedom of movement of the trunk and arms. Category B includes athletes with weakened trunk stabilization caused by, e.g., paraplegia (transverse paraplegia) with a ruptured spinal cord, paralyzed lower limbs and/or minimal hand paresis. Table 1 contains a summary of the basic data on studied fencers in categories A and B.

**Table 1:** Basic data on wheelchair fencers in categories A and B

Fencer	Age	Body height (m)	Body mass (kg)	Sex	Training experience (years)	Condition
*Category A*						
DP	45	1.82	74	M	23	Amputation
NC	33	1.82	70	M	18	Stroke
RT	33	1.81	82	M	7	Paraplegia
KD	25	1.71	68	F	14	Musculoskeletal amputation
MF	30	1.74	69	F	21	Cerebral palsy
RB	36	1.72	70	F	12	Amputation
KK	26	1.53	50	F	5	Myelomeningocele
Mean	32.57	1.74	69.00		14.29	
SD	6.25	0.09	8.93		6.32	
*Category B*						
JG	53	1.8	69	M	6	Multiple sclerosis
AG	18	1.69	60	M	3	Hernia
AC	30	1.67	67	M	11	Paraplegia
KR	35	1.72	73	M	7	Paraplegia
JP	30	1.69	65	F	7	Paraplegia
PH	29	1.52	49	F	8	Paraplegia
JW	18	1.50	50	F	4	Spina bifida
AS	30	1.45	40	F	4	Post-operative lower limb paralysis
SL	30	1.69	55	M	4	Lower limb palsy
Mean	30.33	1.64	58.67		6.00	
SD	9.67	0.11	10.30		2.40	

The study project was approved by the Bioethics Committee of the Medical Chamber (Resolution No. 237 of 13 December 2016), in accordance with the Helsinki Declaration regarding the conduct of clinical trials on humans.

### EMG measurement procedure

The measurement procedures using EMG had been described in detail by Borysiuk et al. [24]. The tests were performed using a 16-channel EMG system (Noraxon, DTS, Desktop Direct Transmission System, Scottsdale, Arizona, USA) with a 16-bit sampling rate of 1500 Hz. Dedicated software was used for the analysis of system data (MyoResearch XP Master Edition for DTS Noraxon), and a wireless transmitter-recorder was used to synchronize the EMG system and transfer the EMG signal directly to the computer. Test procedures, including the placement of electrodes on the fencers’ bodies, were determined according to the SENIAM design. Typical sEMG (hydrogel) Silver/Silver Chloride (Ag/AgCl) electrodes were used in the study.

All participants signed a consent form and gave their permission for publication of the study results. EMG was measured from the latissimus dorsi (right/left) and oblique abdominal (right/left) muscles.

### Controlled movement tasks

The analysis of coherence of the trunk muscles was carried out in a controlled movement, the fencers were in the wheelchair position as shown in previous reports [24], i.e. in a typical fencing position.

#### Preparatory position

During the bout, the fencer assumed a sitting position on the wheelchair attached to the platform, maintaining a distance between the tip of the fencer's weapon and the coach's elbow (flexion arm).

#### The fencer's position awaiting the attack

The fencers sat sideways to each other, with the trunk straight and the weapon held in the dominant hand (limb flexion at the elbow) raised towards the coach. The other hand was fastened with a belt (wrapped on the forearm around the wrist) attached to a special handle on the side of the wheelchairs, which protected the fencer.

#### Position during thrust

At the moment of the thrust, there was a dynamic body abduction in the direction of the thrust on the coach's trunk with a simultaneous rotation of the wrist of the weapon hand. The other hand was secured with a belt. The fencer's movement was caused by the dynamic tension of the trunk and upper limb muscles [24].

After assuming the position, at the command "Ready", the fencer waited for the coach's visual signal. Then the fencer reacted as soon as possible to the movement of the coach's weapon, with an attack and a return to the starting position. This procedure was repeated three times.

### Wavelet coherence

Matlab coherence returns the magnitude-squared wavelet coherence, which is a measure of correlation between signals x and y on the time–frequency plane. Wavelet coherence is useful for analysing nonstationary signals. The coherence is computed using the analytic Morlet wavelet. Wavelet coherence is a measure of correlation between two signals. The wavelet coherence of two time series *x* and *y* is:

$$\frac{{\left| {S(C_{x}^{*} (a,b)C_{y} (a,b))} \right|^{2} }}{{S(|C_{x} (a,b)|^{2} ) \cdot S(|C_{y} (a,b)|^{2} )}}$$

where *C*_*x*_*(a,b)* and *C*_*y*_*(a,b)* denoted the continuous wavelet transforms of *x* and *y* at scales *a* and positions *b*. The superscript* represents the complex conjugate and *S* is a smoothing operator in time and scale. For real-valued time series, the wavelet cross-spectrum is real-valued if a real-valued analyzing wavelet is used, and complex-valued, if a complex-valued analyzing wavelet is applied [27, 28].

**Fig. 1:**
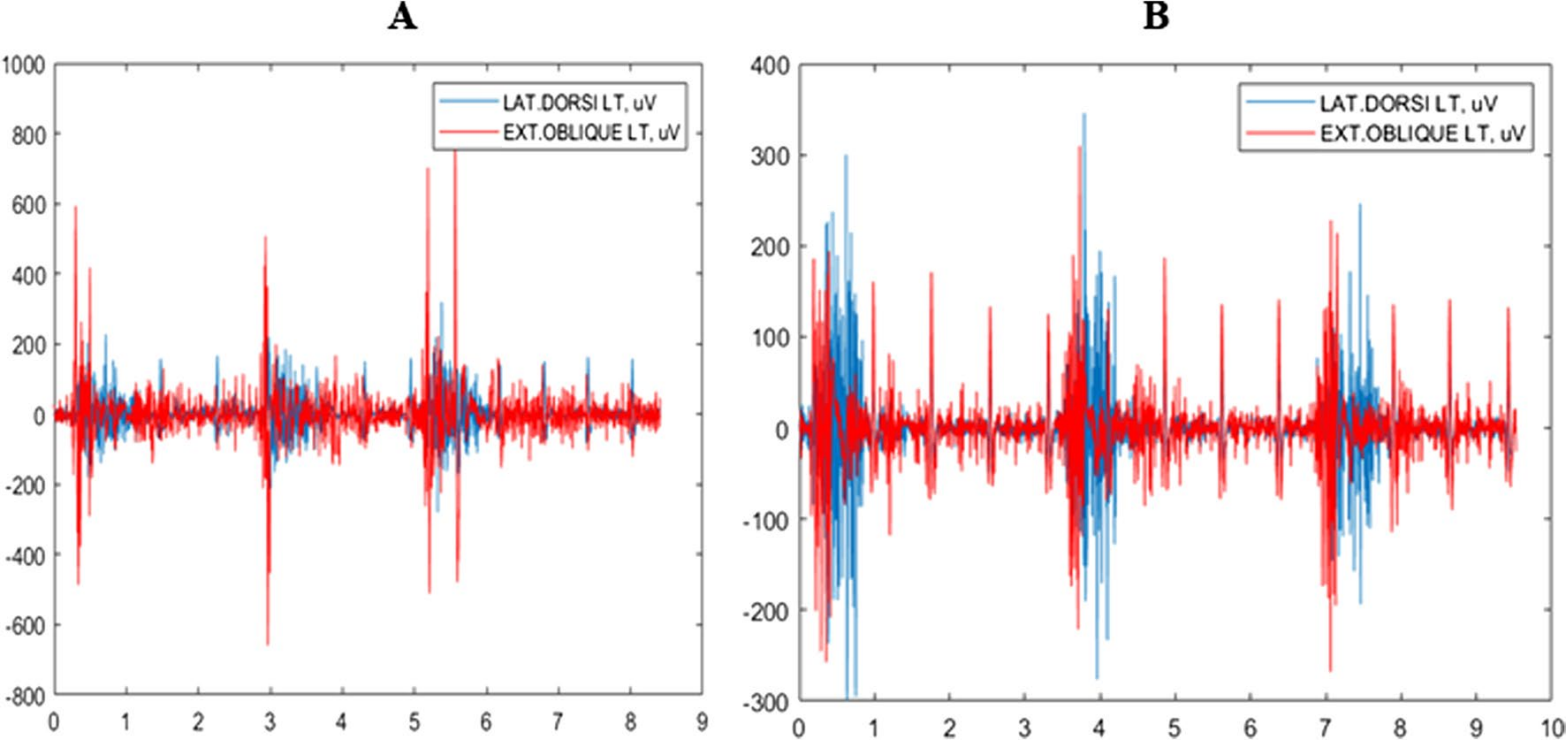
Waveforms of EMG activity in LD and EOA muscles in two fencers (category A, **a**; category B, **b**)

Data on two selected fencers, one from category A and the other from category B, are presented in Fig. 1.

### Data analysis and statistical evaluation

Data analysis consisted of three stages: (1) recording of muscle activation using sEMG, (2) wavelet coherence analysis, and (3) coherence density analysis. The raw data was visually inspected and the signals with disruption or excessive noise were disregarded in the further analysis. The sEMG signals were first full-wave rectified. Such rectification has been demonstrated to enhance the information about the temporal pattern of grouped firing motor units [25, 26]. Coherence is a measure of how closely two EMG signals are related by a linear transformation. Coherence is estimated between 0 and 1, where the value of 1 indicates that the two signals are highly correlated, while the value of 0 means that both signals are independent. EMG signals were recorded with a 10 kHz sampling frequency. Three frequency bands (2–16, 17–30, 31–60 Hz) were selected for the analysis. All coherence points in each specific frequency band from each participant were averaged in order to obtain a large mean coherence value for a given frequency band. The analysis of the coherence of the muscles was performed in various frequency ranges within the 2–60 Hz band. The choice of these bands was based on the standard frequency band 15–30 Hz and the study results that demonstrated the physiological importance of low frequencies and high frequency consistency [9].

### Statistical analysis

Statistical analysis was performed with the use of Statistica 13.1 (StatSoft, Inc., Oklahoma, USA). For abnormal data distribution, non-parametric tests were adopted. The Kruskall Wallis test was used to compare different protocols for controlled muscle activation in the first cohort. The Mann–Whitney test was used to compare the intramuscular coherence of trunk muscles between category A and category B fencers. The level of statistical significance was set at *p* ≤ 0.05.

In order to determine the differences between groups A and B, the coherence for posture stabilizing muscles was to be assessed. For this purpose, two groups of antagonists were selected: the EOA and the LD muscle on the right and the left sides. The analysis covered three frequency ranges, for which the following parameters were calculated: G—share of high 0.9–1 coherence in a given frequency range, and M—mean coherence in a given frequency range.

## Results

The diagram presents the waveform with the raw EMG signal for the LD LT and EOA LT muscles in three lunge attacks performed to a visual stimulus by two selected fencers: category A fencer (Fig. 1a) and category B fencer (Fig. 1b). It can be noted that LD activity appears with a delay in the EOA activity.

**Fig. 2:**
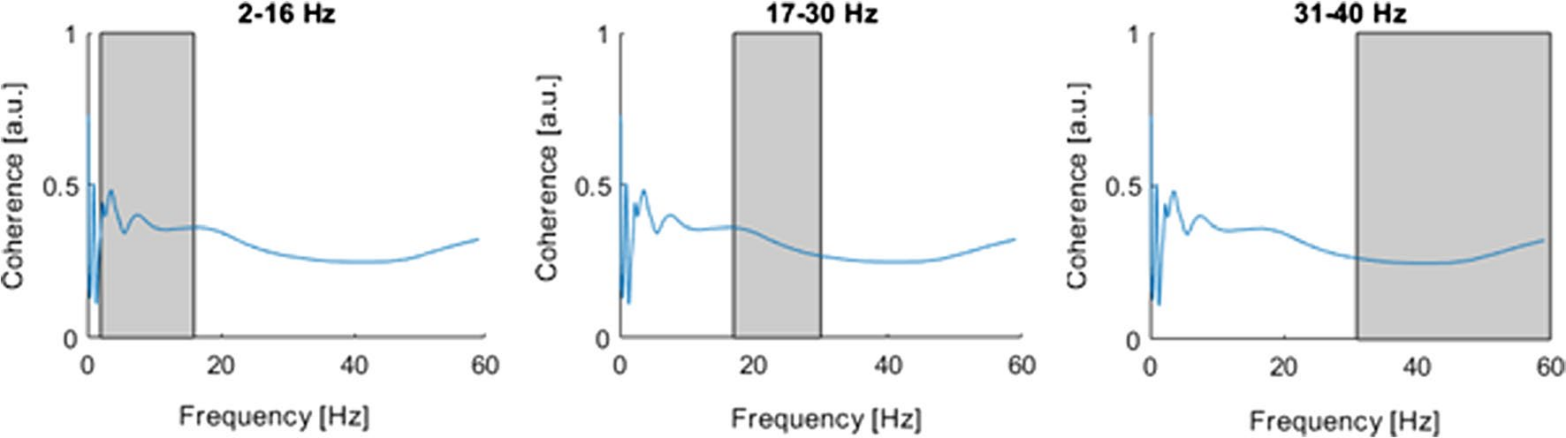
Mean coherence values across time (category A fencer). Mean frequencies within bands: 2–16 Hz, 17–30 Hz, 31–60 Hz. ${\text{M}} = 1 \times 3\quad \quad {\mathbf{0}}.{\mathbf{3953}}\quad {\mathbf{0}}.{\mathbf{3154}}\quad {\mathbf{0}}.{\mathbf{2685}}$. LD, Latisimus Dorsi (RT and LT); EOA, Externus oblique abdominus (RT and LT); RT, right; LT, left

Figure 2 shows Welch averaged coherence estimation determined in three frequency bands. In the case of this fencer, the greatest activity occurred in the B1 band and it amounted to 2–16 Hz. Two frequency modes can be observed in this band. Coherence clearly decreases with increasing frequency and for the B2 bands, and it decreases from 0.32 (B2) to 0.27 for (B3).

**Fig. 3:**
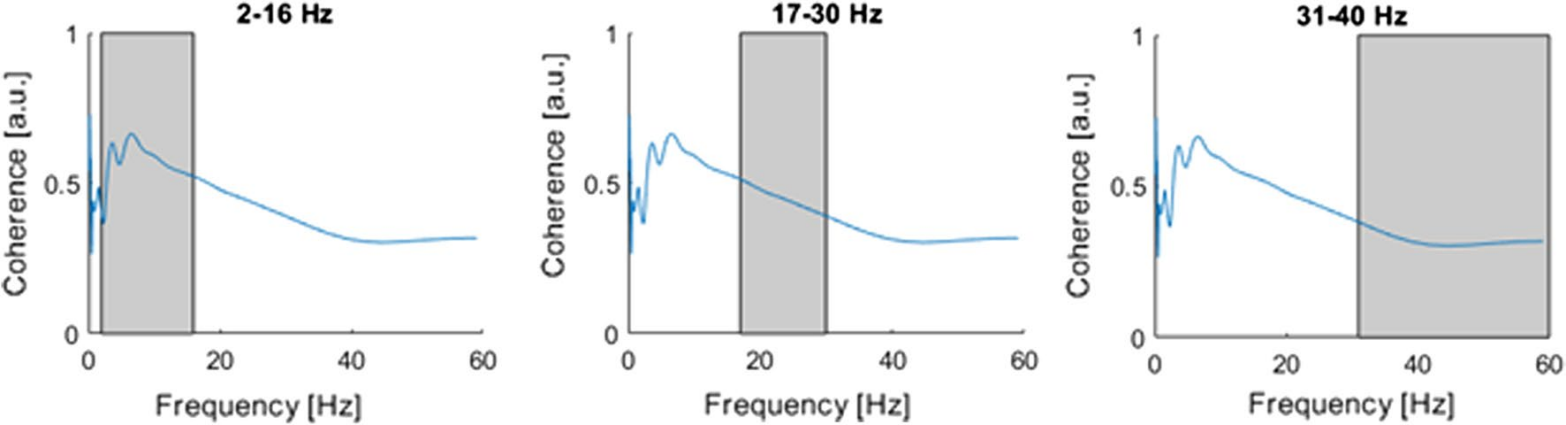
Mean coherence values across time (category B fencer). Mean frequencies within bands: 2–16 Hz, 17–30 Hz, 31–60 Hz. ${\mathbf{M}} = {\mathbf{1}} \times {\mathbf{3}}\quad \quad {\mathbf{0}}.{\mathbf{5649}}\quad {\mathbf{0}}.{\mathbf{4501}}\quad {\mathbf{0}}.{\mathbf{3247}}$. LD, Latisimus Dorsi (RT and LT); EOA, Externus oblique abdominus (RT and LT); RT, right; LT, left

On the other hand, Figs. 2 and 3 demonstrate that the highest activity occurred in the B1 band as well as in the 2–16 Hz band. Again two frequency modes can be observed. The coherence clearly decreases with increasing frequency and for B2 bands, and decreases from 0.45 (B2) to 0.32 for (B3).

As shown in Figs. 2 and 3, for any of the analyzed bands the coherence did not demonstrate values greater than 0.75. This is due to the fact that perhaps coherence did occur, but it took on short-term characteristics, and therefore the averaged analysis does not highlight the short-term occurrence of coherence between muscle activity.

**Fig. 4:**
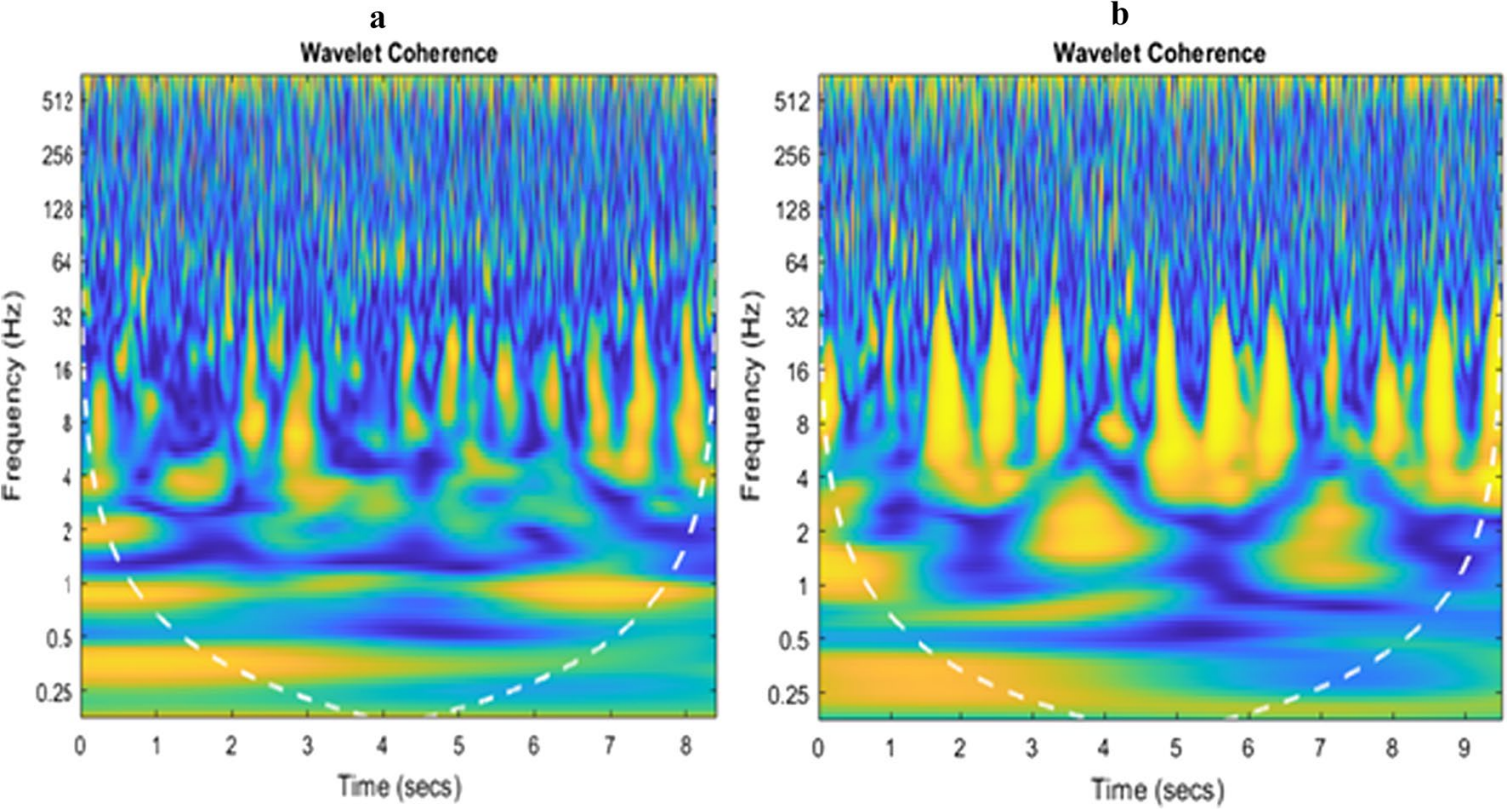
Wavelet cross-coherence between LD and EOA activities (category A, **a**; category B, **b**)

For the A category fencers (Fig. 4a), the highest level of coherence occurs in the range from 8 to 20 Hz. The activities are in the form of short intervals of several dozen ms. Throughout the duration of breaks in activity (return to the waiting for the next movement sequence) there was a coherence in the range of 4–16 Hz. On the other hand, during the highest activity, the coherence has short activity intervals at the level of 8 (about 20 Hz). The category B fencer (Fig. 4b) displayed the greatest coherence in the range of 5 to 15 Hz.

The coherence was in the form of three regions with considerable value for each of the three attempts. It is significant that the coherence increased following the end of the highest muscle activity. The coherence in this respect occured in the intervals between activities. In the case of simultaneous activity, the correlation in the frequency domain occurs at the level of low frequencies, approx. 2–3 Hz, and short-term activity areas at 8 Hz and around 20 Hz. Also a small degree of short-term coherence with a duration of several ms at 60–100 Hz can be observed.

**Fig. 5:**
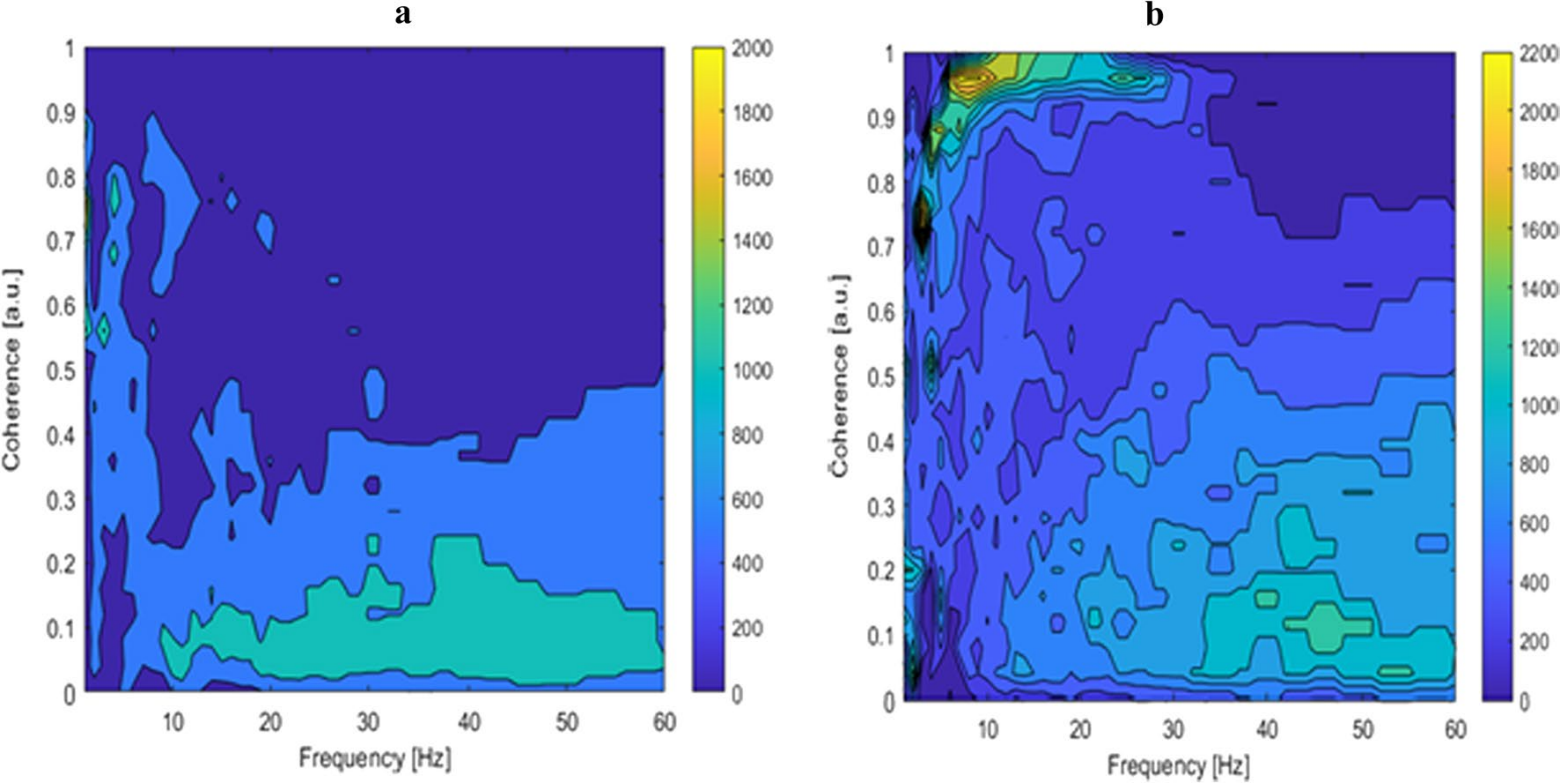
Coherence density (category A, **a**; category B, **b**)

However, the analysis of coherence does not provide more accurate assessment of the variations in time of interaction between the individual frequency ranges. To enable a short-term analysis, magnitude-squared wavelet coherence was determined (Fig. 5a, b), which provides a measure of correlation between sEMG activity in two signals on the time–frequency plane. Wavelet coherence is useful for analyzing nonstationary signals. This distribution makes it possible to determine both the changes in the frequency structure and the changes in this structure over time. The density of coherence was also derived on the basis of wavelet coherence. The category A fencers (Fig. 5a) demonstrated the highest values of coherence density (close to 1) for the frequency range from 4 to 20 Hz, which is confirmed by the results of wavelet coherence distribution. The category B fencers (Fig. 5b) exhibited areas of high coherence in the range of above 0.7, while the highest values were within the range of 5–15 Hz. In particular, the frequency band was divided into 50 ranges. Frequency indices below the cutoff frequency shown on the ordinate axis were determined in each interval. In each case, a histogram of the coherence coefficients in the range from 0 to 1 with a resolution of 0.04 Hz was determined. In this way, a coherence density map revealed the probability of coherence in a given narrow frequency range.

**Fig. 6:**
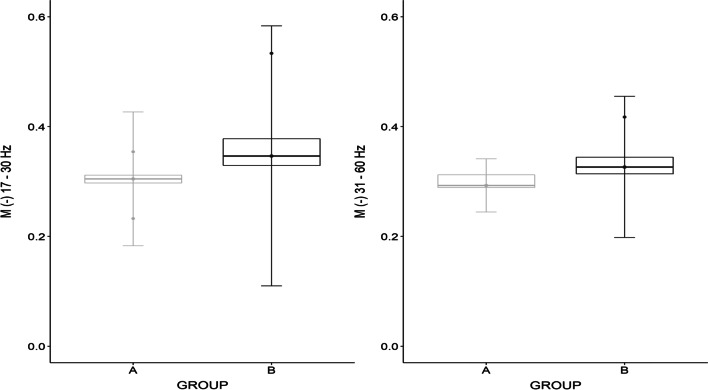
Differences between fencers in categories A and B for muscle pairs LD right/EOA left for the 17–30 and 31–60 Hz bands of parameter M

For the LD right/EOA left muscle pair, statistical significance was demonstrated by the parameter M for the frequency bands 17–30 Hz (W = 9.0, *p* = 0.016) and for 31–60 Hz (W = 12.0, *p* = 0.042). In terms of both parameters, there was a greater coherence for category B fencers (Fig. 6).

In addition, for the LD left E/OA left muscle pair, statistical significance was demonstrated by the G parameter for the frequency range 31–60 Hz W = 8.0, *p* = 0.012, and the M parameter for 17–30 Hz (W = 7.0, *p* = 0.014). There was a greater coherence for the category B fencers.

**Fig. 7:**
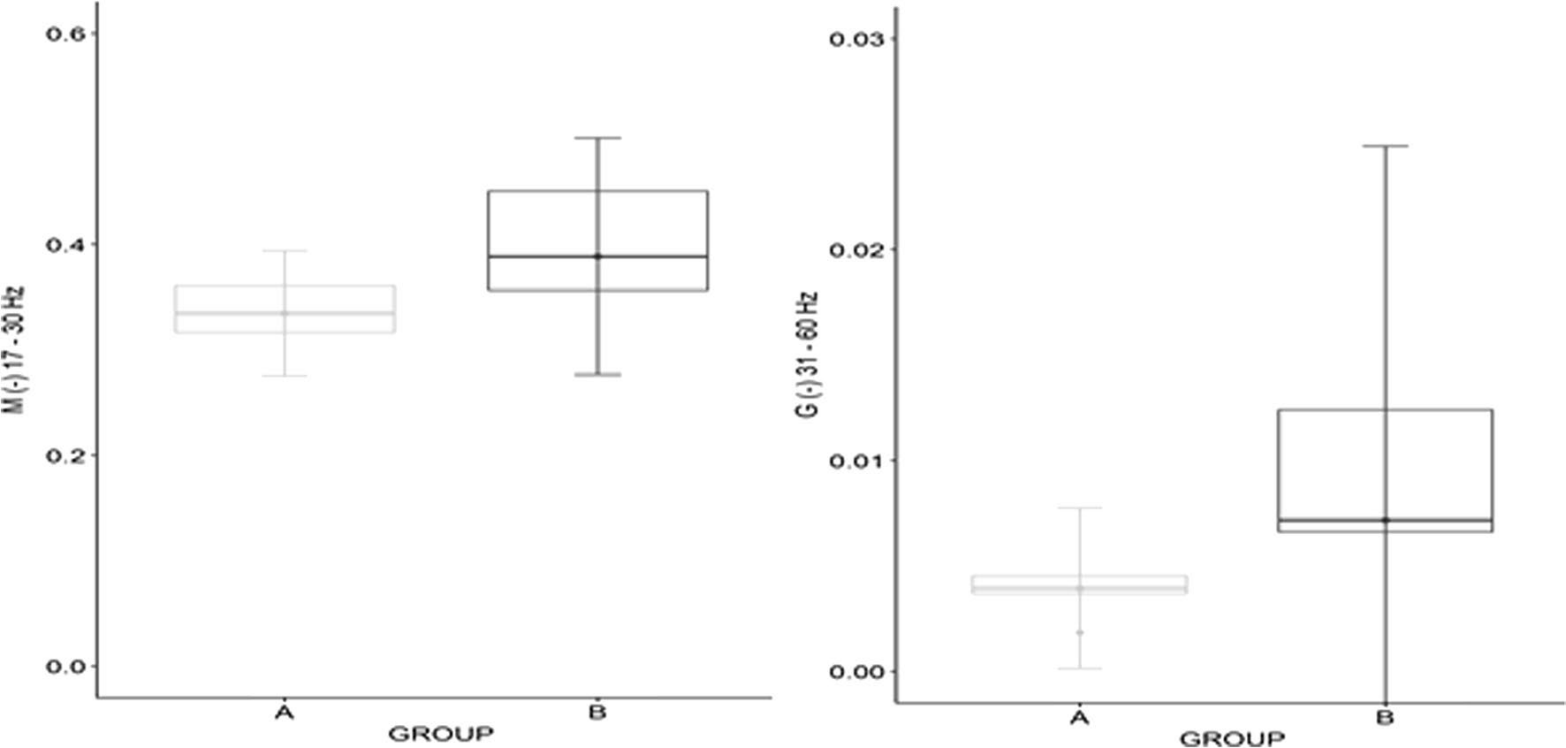
Differences between fencers in categories A and B for the muscle pairs LD left/EOA left for the 31–60 Hz bands of parameter G and 17–30 Hz of parameter M

On the left side, i.e. opposite to the direction of the trunk displacement, a significant coherence was observed at the high frequency level from 31 to 60 Hz, and it was higher in group B (Fig. 7), similarly in the frequency range from 17 to 30 Hz, which potentially demonstrates the postural stability-oriented function of these muscles. The category B fencers B need greater stabilization on the side opposite to the direction of movement, which most likely ensures that they maintain the correct posture when seated in the wheelchair throughout complex motor activities.

Similar relationships were found in the transverse plane (Fig. 6). When the displacement of the torso was analyzed, it turns out that in category B greater coherence was achieved for the left oblique abdominal and the latissimus dorsi muscles, which confirms the stabilization in a sitting position during the performed motor task. During the attack, the fencers tend to lean slightly forward and to the right. The statistical analysis of the antagonistic muscles revealed a greater coherence of the left side of the abdomen and the right side of the back. However, the analysis of the muscles on the opposite side did not demonstrate statistically significant differences for the LD left/EOA right muscle pair. This constitutes evidence in favor of the existence of a pattern that is responsible for postural control in fencers with severe neurological trauma, mainly after spinal cord injury.

**Fig. 8:**
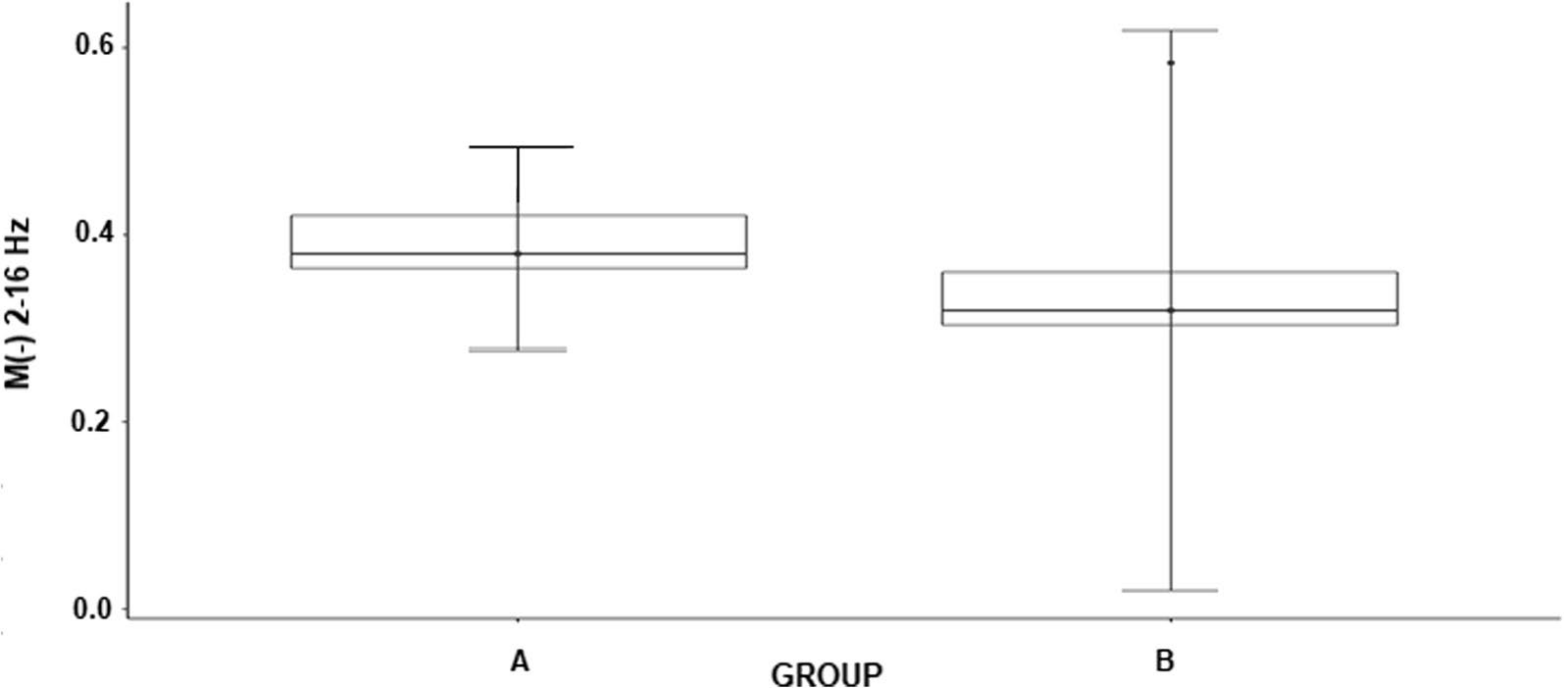
Differences between fencers in categories A and B for muscle pairs LD right/EOA right for the 2–16 Hz of parameter M

For the LD right/EOA muscle pair, statistical significance was demonstrated by parameter M for the frequency range of 2–16 Hz (W = 11.0, *p* = 0.031), and greater coherence was recorded in category A fencers (Fig. 8).

## Discussion

In the present study wavelet analysis was used to determine intermuscular coherence with the purpose of identifying differences in intermuscular synchronization of the main trunk stabilizers in wheelchair fencers. Two groups of antagonist muscles were selected: the oblique abdominal muscle and the latissimus dorsi muscle, and data for the right and the left side muscles was analyzed. The intermuscular EMG-EMG synchronization was estimated for four possible muscle synchronization alternatives. Consequently, the anterior–posterior and lateral movements could be analyzed. The results of the study demonstrate that in Paralympic wheelchair fenecrs, muscles are activated at low frequency levels regardless of category of disability [15, 28, 29]. Moreover, significantly higher rates of coherence were recorded in the category B fencers, at various frequencies. This may indicate that fencers with more severe neurological deficits (category B) require greater trunk muscle stabilization to maintain a stable posture, and skillfully perform difficult motor tasks. This was particularly evident in the area of muscles opposite to the direction of movement (left side). These results may indicate a neuromuscular protective effect of preventing the fencers’ body from shifting excessively in the direction of movement, which could lead to a fall from the wheelchair. In fact, this can be observed at sports events, where the competitor in the euphoria of sports combat, exceeds the limits of stability and, in consequence, is “thrown” out of the wheelchair. The conducted wavelet coherence analysis for selected fencers in categories A and B demonstrated intermuscular synchronization at low frequency levels (8 to 20 Hz for category A fencers, 5–15 Hz for category B fencers). Moreover, in the case of category A fencers (Fig. 4a), in the phase of preparation for an attack, intermuscular synchronization takes the form of short intervals lasting a few dozen ms. However, during the phase of muscle response (attack), the coherence had short activity intervals at the level of 8, at about 20 Hz. For category B fencers (Fig. 4b), the coherence is observed in three large areas for each of the three trials. It is significant that the coherence increases after the cessation of the highest activity (attack), even up to several ms in the range of 60–100 Hz. Similar relationships were reported in two studies that examined the sEMG activity in the static muscles of the trunk, carried out among groups of participants with and without spinal cord injury, and a greater level of activity was found in individuals with spinal cord injury in comparison to the control group without disability. People with more severe spinal damage had to generate more muscle strength to control the trunk during both sitting and anterior–posterior movement patterns [28].

The results suggest the existence of a low frequency range of muscle activity stimulation in fencers with spinal cord injury, which may indicate the spinal circuit origin of this activity; however, studies had confirmed that this phenomenon can be generated by spinal circuits in a situation where descending drive is abolished or severely reduced [30, 31]. Intermuscular coherence at 3–10 Hz has also been reported in myoclonus dystonia, which is a movement disorder with a genetic basis, in spinal cord injured subjects with incomplete lesions in whom significant coherence at lower frequencies (10–16 Hz) was found during brief voluntary contractions. This suggests the possibility of a subtle interplay between the central and peripheral circuits that can either cancel, or possibly generate, low frequency activity [15, 31].

Wang et al. recommended the use of sEMG as an assessment tool for improving the comparability and interpretability of trunk muscle activity in SCI therapeutic strategies [28]. Surface EMG is an informative complement to current clinical testing and can capture the residual motor command in great detail, also in muscles below the level of injury with seemingly absent motor activities. Nonetheless, recent studies have been constrained to amplitude-based sEMG analyses, and there are opportunities to more broadly characterize the time- and frequency-domain properties of the signal as well as to take fuller advantage of high-density EMG techniques. We recommend the incorporation of a broader range of signal properties into the neurophysiological post-SCI assessment and the development of a greater understanding of the relationship between these sEMG properties and underlying physiology. Enhanced sEMG analysis could contribute to the understanding of the mechanisms of change following neuromodulation or exercise therapy [29].

## Conclusions

Due to the fact that integrity of the motor and sensory neurological systems is impaired in individuals with neurological deficits, the demands on the trunk are increased in them. These findings should prompt further investigations into trunk muscle function in wheelchair athletes and highlight the importance of including sEMG tests for trunk muscles in the process of training of people with neurological deficits. Moreover, wavelet analysis is an efficient tool for examining muscle activation in the lower frequency range, which is otherwise undetectable in traditional correlation analysis.

## Data Availability

The datasets analyzed in the study are available from the corresponding author.
